# Muscle fitness and its association with body mass index in children and adolescents aged 7–18 years in China: a cross-sectional study

**DOI:** 10.1186/s12887-019-1477-8

**Published:** 2019-04-10

**Authors:** Huijing He, Li Pan, Jianwei Du, Feng Liu, Yuming Jin, Jingang Ma, Li Wang, Pengben Jia, Zhiping Hu, Guangliang Shan

**Affiliations:** 1 Department of Epidemiology and Statistics, Institute of Basic Medical Sciences, Chinese Academy of Medical Sciences, 5 Dongdansantiao, Dongcheng District, Beijing, 100005 China; 2 Department of Epidemiology and Statistics, School of Basic Medicine, Peking Union Medical College, 5 Dongdansantiao, Dongcheng District, Beijing, 100005 China; 3 Hainan Provincial Center for Disease Control and Prevention, Haikou, 570203 Hainan Province China; 4 Shaanxi Provincial Center for Disease Control and Prevention, Xi’an, 710054 Shaanxi Province China

**Keywords:** Muscle fitness, Body mass index, Children, Adolescents, Health, China

## Abstract

**Background:**

The present study was the first one aimed to investigate the current muscle fitness and its associated factors among children and adolescents in mainland China.

**Methods:**

From Nov 2013 to Jul 2014, 2283 children and adolescents aged 7–18 were recruited in Hainan and Shaanxi Provinces in China by cross-sectional design. Information on anthropometry and muscle fitness, measured by hand grip strength (GS), vertical jump (VJ) and sit-and-reach (SR), were collected. Analysis of covariance was performed by using general linear regression models to identify the association between BMI and muscle fitness.

**Results:**

The means of GS, VJ and SR in boys were 22.30 ± 11.55 kg, 22.93 ± 6.80 cm and 3.58 ± 7.31 cm, respectively, and in girls were 16.61 ± 6.87 kg, 18.11 ± 4.08 cm and 7.18 ± 5.72 cm, respectively. GS (from 8.26 kg in the 7–8-year-old group to 27.91 kg in the 17–18 group) and SR (from 1.75 cm in the-8-year-old group to 10.12 cm in the 17–18 group) increased with age (both *p* for trend < 0.001). Boys had higher GS and VJ, but significantly lower SR than girls in each age group (*p* < 0.001). After adjusting for age, sex, residential areas and study regions, GS increased with elevated BMI (compared with normal weight group, the regression coefficient for thinness and overweight/obesity were − 2.997(95%CI: −3.693 to − 2.301) and 1.220 (95%CI: 0.285 to 2.155), respectively. With the *p* values less than 0.001 and 0.011, respectively). For VJ, there was no difference found between normal weight group and overweight/obesity group (*p* = 0.550), but the thinness group had the lowest performance (regression coefficient = − 2.681, 95%CI from − 3.965 to − 1.397, *p* < 0.001). For SR, compared with normal weight group, the regression coefficients for thinness and overweight/obesity were − 1.313(95%CI: −2.228 to − 0.399) and − 1.623(95%CI: −3.216 to − 0.030) respectively, both *p* < 0.05.

**Conclusions:**

Increased body weight may have a positive association with isometric muscle strength measured by grip strength, but a negative one with strength of lifting the body. Sex difference was also found in the performance of flexibility.

**Electronic supplementary material:**

The online version of this article (10.1186/s12887-019-1477-8) contains supplementary material, which is available to authorized users.

## Background

Muscle fitness is an important aspect of physical fitness and health status [1]. It can be defined as the maximal force or tension that a muscle or a group of muscles could generate at a specified velocity [2]. A decrease of muscle fitness may result in functional limitations [3] and musculoskeletal components were found inversely associated with metabolic risk [4, 5]. There was substantial evidence that indicated that youth muscle fitness (MF) was an important marker of cardiovascular disease (CVD) risk factors [6–8], as well as CVD events in children and adolescents [9]. Therefore, MF in juveniles may be a valuable assessor of health risk factors.

Vertical jump (VJ) has been commonly used to assess muscular power in the lower limbs and can often provide information regarding functional capacity [10]. There were previous studies that investigated the relationship between vertical jump and anthropometric characteristics or established normative data [2, 11–13]. However, because all of these studies were not conducted in Asian children or adolescents, data on youths in mainland China is sparse.

The sit-and-reach (SR) test is a field test used to measure hamstring and lower back flexibility [14]. Hand grip strength (GS) is a measurement for upper body muscle strength [15]. GS can be used as an indicator for an individual’s general muscle strength [1]. Vertical jump, hand grip strength and sit-and-reach were considered indexes for muscle fitness in the present study. Since the ability to perform short-term maximal exercise varies between populations, it is important to investigate population specific data on MF.

Age, gender, morphological and metabolic factors have been found as determinants in anaerobic performance [16]. Moreover, there were previous studies that suggested that body mass index (BMI) was associated with muscle fitness [17, 18], and that increasing weight may be related to greater performance on muscle fitness tests. Geographical, socioeconomic and sexual disparities in health-related physical fitness and BMI were also observed [19, 20]. Child overweight and obesity has risen in middle- and low- income countries [21]. Accompanied with rapid socioeconomic progress in China in the past decades, the prevalence of overweight and obesity in children and adolescents also increased and was believed to be associated with urbanization [22]. However, data on MF and its relationship with BMI in Chinese children and adolescents is sparse.

Therefore, the objective of this study was to use the data derived from a community-based cross-sectional study to provide information on the status of MF, as well as its relationship with body weight, measured by BMI, among children and adolescents aged 7–18 by gender and age groups. To the best of our knowledge, this was the first study in mainland China to explore the current situation of MF performance and its relationship with BMI in children and adolescents.

## Methods

### Study design

**Fig. 1:**
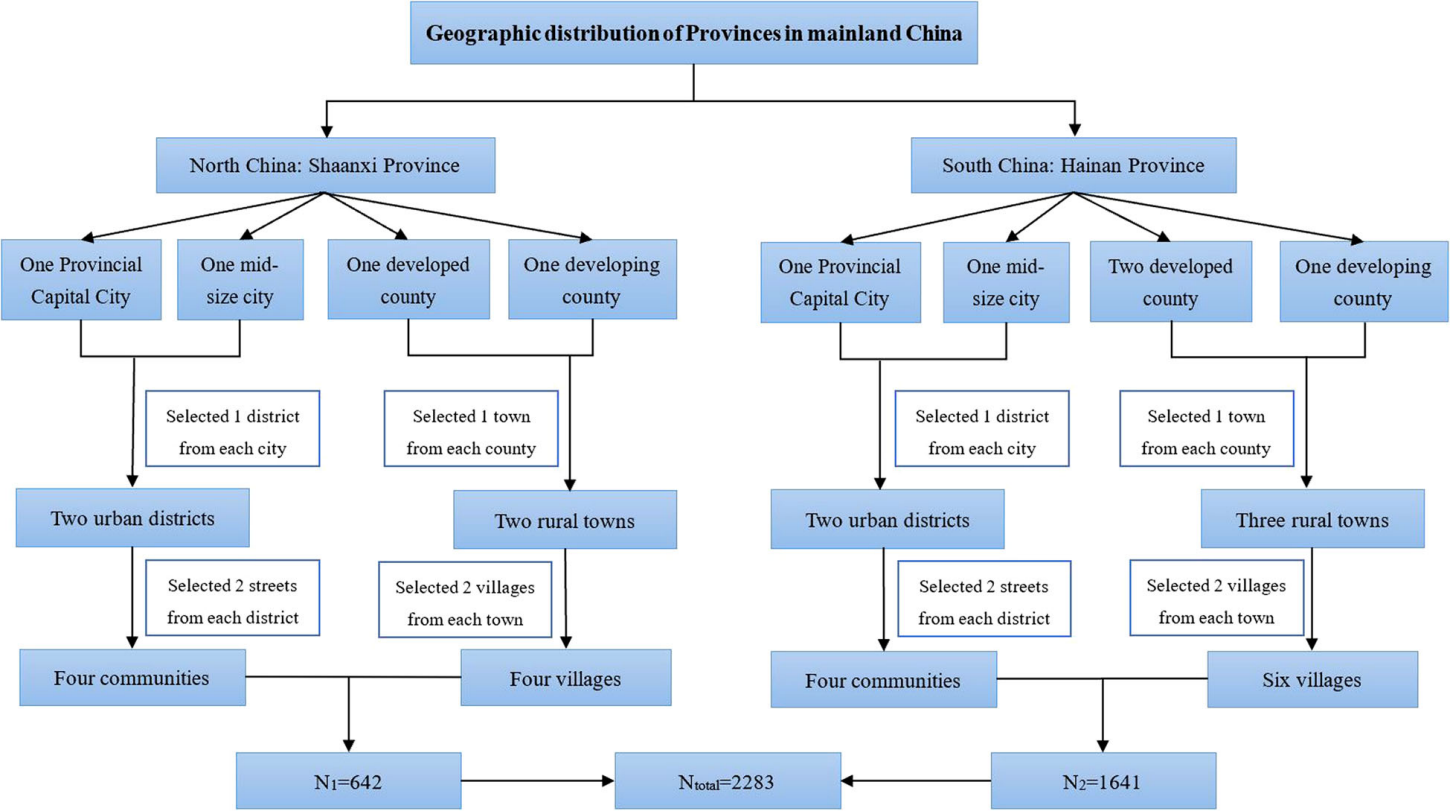
Flow chart of the sampling method for selecting children and adolescents aged 7–18 from Nov 2013 to July 2014 in Northwest and South China

Cross-sectional design was used in the present study. From Nov 2013 to Jul 2014, a multi-stage stratified cluster sampling method was used to select subjects (see Fig. 1: The flow-chart of the sampling method). In the first stage, Shaanxi Province in Northwest China and Hainan Province in South China were selected. In the second stage, two cities and two counties were selected from each province based on their economic status measured by local gross domestic product (GDP). In the third stage, districts were selected from cities, and rural townships were selected from counties. In the final stage, communities were selected from districts in urban areas, whereas villages were selected from townships in rural areas. All children and adolescents lived in the selected districts and villages were all invited to participant in the study. To guarantee a representative sample, after each day’s field work, the age-, sex- and urbanization-stratified participants proportion would be calculated and compared with the local population proportion. The enrollment proportion for the next day’s participants would be correspondingly modified if there was a slight deviation.

### Subjects

A total of 2283 children and adolescents living in Hainan (South China) and Shaanxi (Northwest China) Province participated in the study. Children and adolescents aged 7–18 who were residents in the selected areas and who had lived in the current residence for at least 1 year were eligible to participate. Ethical approval was obtained from the Bioethical Committee of Institute of Basic Medical Sciences, Chinese Academy of Medical Sciences. All parents of the participants provided written informed consent before the survey.

### Procedures

A standard questionnaire was developed to conduct face-to-face interview. Demographic information, such as sex, age and residential areas, was obtained through the parents of the subjects. Physical examinations were conducted and collected information on anthropometry and MF. Anthropometry included stature and weight. Measurements on MF consisted of hand grip strength, vertical jump and sit-and-reach. Before the survey, all interviewers and technicians completed a training program that guaranteed their ability for using specific tools and methods.

Stature was measured to nearest 0.1 cm using a fixed stadiometer. Weight was measured by body composition analyzer (TANITA BC-420, Japan), with the accuracy to the decimal level. During the anthropometric measurements, participants wore light clothing and were barefoot. BMI was calculated as weight in kilograms divided by the square of stature in meters (kg/m^2^).

Vertical jump was evaluated by the Squat Jump (SJ). During the SJ, participants were instructed to sink and to hold a squat position for 3 s. On the count of three, subjects were asked to jump as high as possible. Jump with no sinking or countermovement prior to the execution was considered as a successful try. Hand grip strength of the predominant hand of each participant was measured two times using Jamar Hydraulic Hand Evaluation Kit (JAMAR, UK) in a standing position. In the sit-and-reach test, subjects were examined wearing light clothes and no shoes. The test was performed twice consecutively, with 30 s rest between tests. Subjects assumed a long-sitting position on the board, kept the knees fully extended and feet dorsiflexed and positioned flat against the foot platform. The fingertips were placed together and adjacent to the block that laid along the scale. The subject’s hands pushed the block forward the scale as far as possible and the scale measurement was recorded. Vertical jump, hand grip strength and sit-and-reach tests were all requested to perform twice and the larger one of each item was analyzed.

Thinness, normal weight, overweight and obesity were defined according to World Health Organization (WHO) ‘s criteria for children and adolescents 5–19 years old: thinness was BMI-for-age lower than 2 standard deviations below the WHO Growth Reference median; overweight was BMI-for-age greater than 1 standard deviation above the WHO Growth Reference median; and obesity was greater than 2 standard deviations above the WHO Growth Reference median [23].

### Statistical analyses

All statistical procedures were performed using SAS 9.4 (SAS Institute Inc. Cary, NC, USA). Summary results were presented as mean (standard deviation, SD) for continuous data and number (percentage, %) for categorical data. Data from boys and girls were analyzed separately. Mean, standard deviation (SD), median and interquartile range were reported for all MF tests by gender and age groups because of the non-normal distribution of the results.

Chi-square tests or Student’s t-test (or Wilcoxon sign test) were used to compare characteristics of participants in the analytic sample (*n* = 2283). Comparison among age groups was analyzed using ANOVA or Kruskal-Wallis test. Cochran–Armitage test was used to analyze the trend among age groups and BMI groups. As it was expected that the measures of MF in this study were possibly correlated, we further calculated correlation coefficients of the three indexes for MF using Pearson and Spearman correlation analyses. Since there were limited number of obesity in both boys and girls, obesity was integrated with overweight as one category, presented as overweight/obese in the results section.

Two-way ANCOVA was performed by using general linear regression models (GLMs) to identify the association between BMI and muscle fitness (GS, VJ and SR). In the sensitivity analyses, quantile regression models were used to compare the results yielded by GLM. Sub-group analyses were conducted within age-groups to detect the possible role of age on the association between BMI and muscle fitness.

## Results

### Demographic and anthropometric characteristics of participants

The analyses for this study were based on 2283 children and adolescents (1032 boys and 1251 girls) aged 7–18 who participated in the muscle fitness tests and were classified in three BMI categories. The demographic and anthropometric characteristics stratified by sex were presented in Table 1. Boys had a higher average age and proportion of living in rural areas, and were more recruited in Shaanxi Provinces than that of girls.

**Table 1:** Baseline characteristics of children and adolescents. N and percentage (%) for categorical data and mean and standard deviation for continuous data

	Boys (*n* = 1032)		Girls (*n* = 1251)		Total (*n* = 2283)	
Age^*^, year	13.36	3.11	13.77	3.36	13.58	3.26
Age-group^*^						
7–8	73	7.07	94	7.51	167	7.31
9	97	9.40	126	10.07	223	9.77
10	115	11.14	128	10.23	243	10.64
11	111	10.76	116	9.27	227	9.94
12	119	11.53	104	8.31	223	9.77
13	103	9.98	80	6.39	183	8.02
14	78	7.56	62	4.96	140	6.13
15	74	7.17	98	7.83	172	7.53
16	82	7.95	140	11.19	222	9.72
17	78	7.56	151	12.07	229	10.03
18	102	9.88	152	12.15	254	11.13
Residential areas^*^						
Urban	514	49.81	702	56.12	1216	53.26
Rural	516	50.00	549	43.88	1065	46.65
Study sites^*^						
Shaanxi Province	333	32.27	309	24.70	642	28.12
Hainan Province	699	67.73	942	75.30	1641	71.88
Stature^*^, cm	152.55	16.13	148.67	12.19	150.43	14.24
Weight^*^, kg	42.11	13.87	39.2	10.63	40.51	12.28
BMI, kg/m^2^	17.58	3.16	17.37	2.78	17.47	2.96
Vertical jump^*^, cm	22.93	6.80	18.11	4.08	20.18	5.92
Hand grip strength^*^, kg	22.30	11.55	16.61	6.87	19.17	9.69
Sit-and-reach^*^, cm	3.58	7.31	7.33	7.18	5.72	7.47

^*^p < 0.05 for the comparison between boys and girls

### The distribution of body mass index and its associated factors

82.29% of boys and girls were normal weight, 12.66% were underweight and 7.05% were overweight/obese. The prevalence of underweight, normal weight and overweight/obesity in boys were 14.24, 75.78 and 9.98%, respectively, and in girls were 11.35, 84.01 and 4.64%, respectively. The sex-specific prevalence of thinness and overweight/obesity were presented in Additional file 1: Table S1. The results of multi-variable logistic regression models indicated that, boys (OR = 1.371, 95% CI: 1.065–1.764), urban residence (OR = 0.447, 95% CI: 0.336–0.594) and living in Shaanxi Province (OR = 0.534, 95% CI: 0.387–0.737) were associated with thinness (reference group = normal weight). This participants showed that who were male, living in urban areas and recruited in Shaanxi Province were more likely to be overweight/obese, with ORs (95% CI) of 2.260 (1.594–3.203), 3.118 (2.164–4.492) and 3.668 (2.615–5.202), respectively. In contrast, age was found inversely associated with overweight/obesity with the OR (95% CI) of 0.816 (0.769–0.865) (Additional file 1: Table S5).

### Performance of muscle fitness and its associations with body mass index

#### Performance on the hand grip strength test

**Fig. 2:**
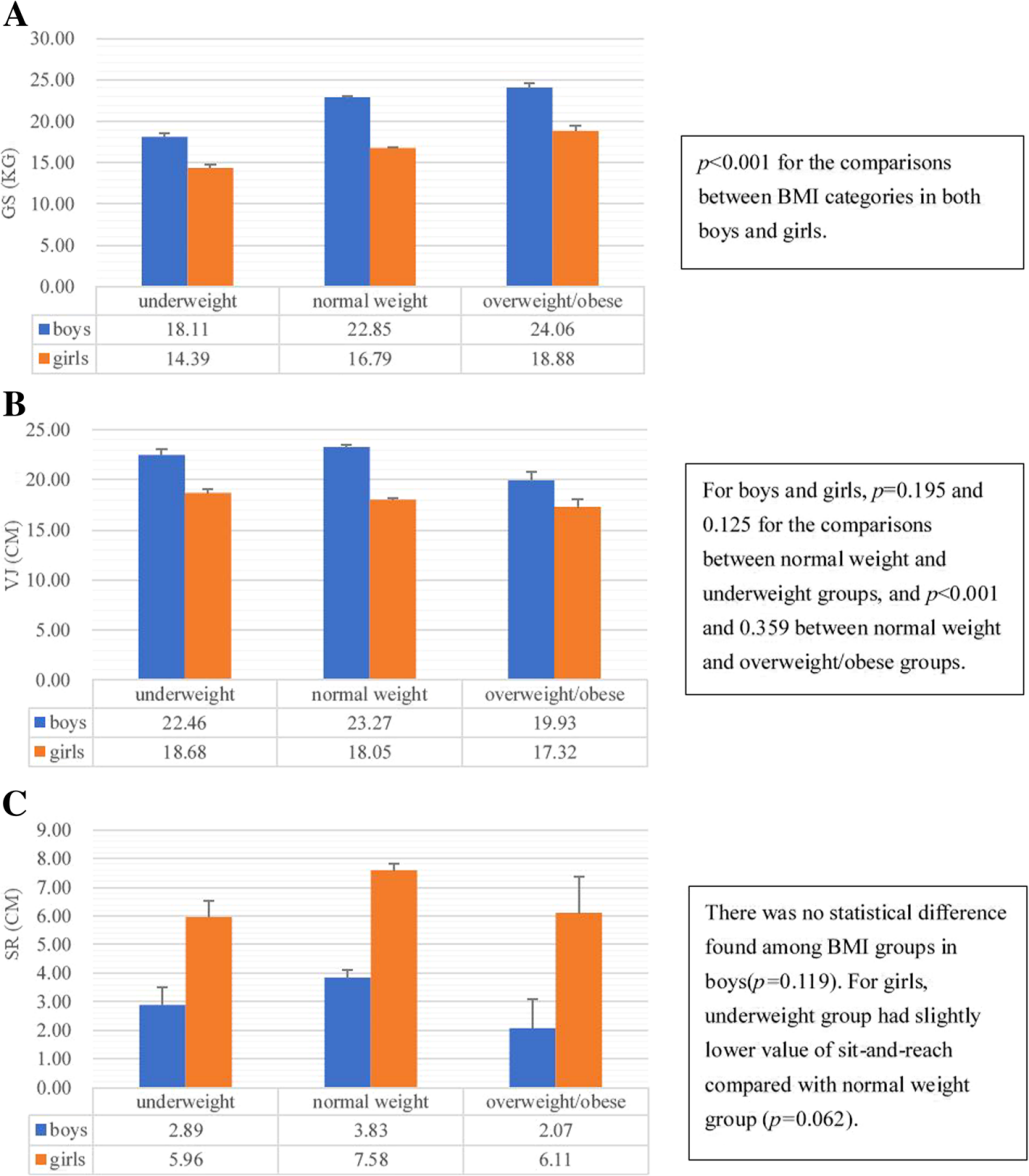
Adjusted means and standard errors of hand grip strength, vertical jump and sit-and-reach across sex and BMI categories in children and adolescents aged 7–18 in China. Adjusted covariates included age, urbanization and geographic areas

The means of GS in boys and girls were 22.30 ± 11.55 kg and 16.61 ± 6.87 kg, respectively. Positive age dependent linear trends were observed in both sexes (Table 2, both *p* < 0.001). The means of GS increased from 8.26 kg in the 7–8-year-old group to 27.91 kg in the 17–18-year-old group. Before adjusting for age, the normal weight group had the highest GS in both sexes. However, the age-, urbanization-and geographic-adjusted means of GS indicated a significantly increasing trend of GS with elevated BMI categories, from 18.11 kg to 24.06 kg in boys and 14.39 kg to 18.88 kg in girls, in which the overweight/obese group had the highest GS in both sexes. The comparisons of the adjusted means of GS among BMI groups were presented separately by sex in Fig. 2a. GLMs also supported the idea that increased BMI was positively associated with GS performance (Table 3). After adjusting for age, sex, residential areas and study regions, BMI was found associated with GS (Table 3). Before BMI was considered in the GLM, study regions (Shaanxi Province) was found associated with GS (*P* = 0.002), but the association became nonsignificant after adjustment for BMI (*P* = 0.086, Table 3).

**Table 2:** The means and medians of hand grip strength, vertical jump and sit-and-reach in age groups across sex among children and adolescents aged 7–18 in China, 2014

Age		GS (kg)					VJ (cm)					SR (cm)			
	n	Mean	SD	Median	IQR	n	mean	SD	Median	IQR	n	mean	SD	Median	IQR
Boys															
7–8	73	9.40	2.49	10.00	3.00	42	18.69	3.22	18.65	5.00	45	0.20	5.66	− 0.60	7.70
9–10	208	11.88	3.29	12.00	4.00	142	18.58	4.09	18.25	6.10	149	0.60	5.79	1.50	7.40
11–12	223	15.62	5.19	15.00	6.00	157	21.46	5.52	21.30	7.60	165	1.01	5.88	1.80	7.80
13–14	178	24.29	7.19	24.00	10.00	119	23.81	6.23	22.90	7.70	122	3.91	6.86	3.90	8.80
15–16	155	32.79	7.61	32.00	9.00	93	25.76	7.17	25.30	9.30	94	7.89	7.52	7.65	10.50
17–18	179	36.91 †	6.75	36.00	9.00	119	28.47	7.08	28.40	9.10	120	8.34	7.51	8.10	8.55
Overall	1016	22.30	11.55	19.00	20.00	672	22.93	6.80	21.95	9.05	695	3.58	7.31	3.30	9.30
Girls															
7–8	93	7.36^*^	2.14	8.00	2.00	42	17.06	4.00	16.50	5.10	67	2.79^*^	5.19	2.50	6.40
9–10	252	9.70^*^	3.08	10.00	4.00	142	17.48	4.08	17.00	5.50	183	3.48^*^	5.75	3.40	7.20
11–12	218	14.47^*^	4.80	14.00	7.00	157	19.15^*^	4.03	19.20	4.50	162	4.72^*^	6.19	4.35	7.30
13–14	142	18.54^*^	4.47	18.00	6.00	119	18.51^*^	4.13	18.35	5.65	102	7.48^*^	6.81	7.85	9.60
15–16	238	20.79^*^	4.53	21.00	6.00	93	18.11^*^	4.09	18.00	5.20	180	10.34^*^	6.70	10.45	8.45
17–18	302	22.58^*^	4.76	22.00	5.00	119	17.98^*^	3.96	17.95	5.80	236	11.02^*^	6.89	11.80	9.25
Overall	1245	16.61^*^†	6.87	17.00	11.00	672	18.11^*^	4.08	18.00	5.50	930	7.33^*^†	7.18	7.20	9.80

*SD* standard deviation, *IQR* interquartile range

^*^p < 0.001 for the comparison between boys and girls

†*p* < 0.001 for the linear trend tests using Cochran–Armitage method

**Table 3:** The association between BMI and hand grip strength, vertical jump and sit-and-reach in children and adolescents aged 7–18 in China, 2014

Hand grip strength	Model 1^a^					Model 2				
	*B*	*SE*	95% *CI*		*p*	*B*	*SE*	95% *CI*		*p*
Age	2.253	0.038	2.178	2.328	< 0.001	2.275	0.038	2.200	2.350	< 0.001
Sex (ref = girls)	6.546	0.239	6.077	7.015	< 0.001	6.580	0.236	6.117	7.043	< 0.001
Residential areas (ref = rural)	0.196	0.255	−0.304	0.695	0.442	0.203	0.256	−0.704	0.298	0.427
Shaanxi (ref = Hainan)	0.822	0.270	0.293	1.352	0.002	0.467	0.272	−0.065	0.999	0.086
Thinness (ref = normal weight)	–	–	–	–	–	−2.997	0.355	−3.693	−2.301	< 0.001
Overweight/obesity (ref = normal weight)	–	–	–	–	–	1.220	0.477	0.285	2.155	0.011
Vertical jump										
Age	0.475	0.046	0.385	0.564	< 0.001	0.442	0.046	0.351	0.532	< 0.001
Sex (ref = girls)	5.107	0.267	4.584	5.631	< 0.001	5.179	0.267	4.656	5.701	< 0.001
Residential areas (ref = rural)	−0.145	0.304	−0.740	0.450	0.632	0.109	0.309	−0.496	0.715	0.723
Thinness (ref = normal weight)	–	–	–	–	–	0.230	0.385	−0.524	0.985	0.550
Overweight/obesity (ref = normal weight)	–	–	–	–	–	−2.681	0.655	−3.965	−1.397	< 0.001
Sit-and-reach										
Age	0.998	0.056	0.888	1.108	< 0.001	0.981	0.057	0.870	1.092	< 0.001
Sex (ref = girls)	−3.193	0.327	−3.834	−2.553	< 0.001	− 3.114	0.327	− 3.754	− 2.473	< 0.001
Residential areas (ref = rural)	0.180	0.371	−0.907	0.548	0.628	−0.177	0.379	−0.921	0.566	0.640
Thinness (ref = normal weight)	–	–	–	–	–	−1.313	0.467	−2.228	−0.399	0.005
Overweight/obesity (ref = normal weight)	–	–	–	–	–	−1.623	0.813	−3.216	−0.030	0.046

*BMI* body mass index (kg/m^2^), *B* regression coefficient, *SE* standard error of regression coefficient, *CI* confidence interval

^a^Model 1: adjusted for age, sex, residential areas and study sites; Model 2: adjusted for age, sex, residential areas, study sites (if applicable) and BMI. Age was analyzed as continuous data; sex, BMI, residential areas and study sites were set as dummy variables

#### Performance on the vertical jump test

Similar to GS, sex difference was also found in VJ performance, where the means of vertical jump in boys (22.93 ± 6.80 cm) were higher than that of girls (18.11 ± 4.08 cm). Different from hand grip strength, the variations of VJ among age groups were not as much as that of GS, ranging from 17.74 cm in the youngest group to 21.56 cm in the 15–16-year-old group. VJ performance varied by age groups in both sex (both *p* < 0.01). But for boys, VJ seemed increased with age, and for girls, the VJ peak was in the 11–14 age group. Boys had greater performance on VJ than girls in the age groups beyond 10 years old (Table 2).

In contrast with GS, overweight/obese youth in both boys and girls had the lowest value of VJ, the age-, urbanization-and geographic-adjusted means of which were 19.93 cm and 17.32 cm, respectively (Fig. 2b). The linear regression model also revealed that BMI categories were inversely associated with VJ (Table 3). Moreover, sex disparity was found in the relationship between VJ and BMI when comparing the adjusted means of MF performance. In boys, underweight and normal weight groups had greater VJ values (22.46 cm and 23.27 cm, respectively) than overweight/obese group (19.93 cm) (both *p* < 0.001), but no difference between underweight and normal weight children and adolescents (both *p* values more than 0.1, see Fig. 2b). However, in girls, the VJ performance had no statistical difference among the three BMI groups (all *p* > 0.05, Fig. 2b).

#### Performance on the sit-and-reach test

Significant sex difference was observed on the performance of sit-and-reach test. The means of SR in boys and girls were 3.58 cm and 7.33 cm, respectively. In every age group, girls had higher SR value than that of the boys (Table 2). Similar to GS, there was an increasing trend with age in both sexes (*p* < 0.001). The GLM indicated that lean youth may have lower value on SR performance. The age-, urbanization-and geographic-adjusted means of SR in each BMI category, stratified by sex, also revealed a positive association between BMI and SR performance, with an exception of no statistical significance in male youth.

#### The correlation among muscle strength tests

**Fig. 3:**
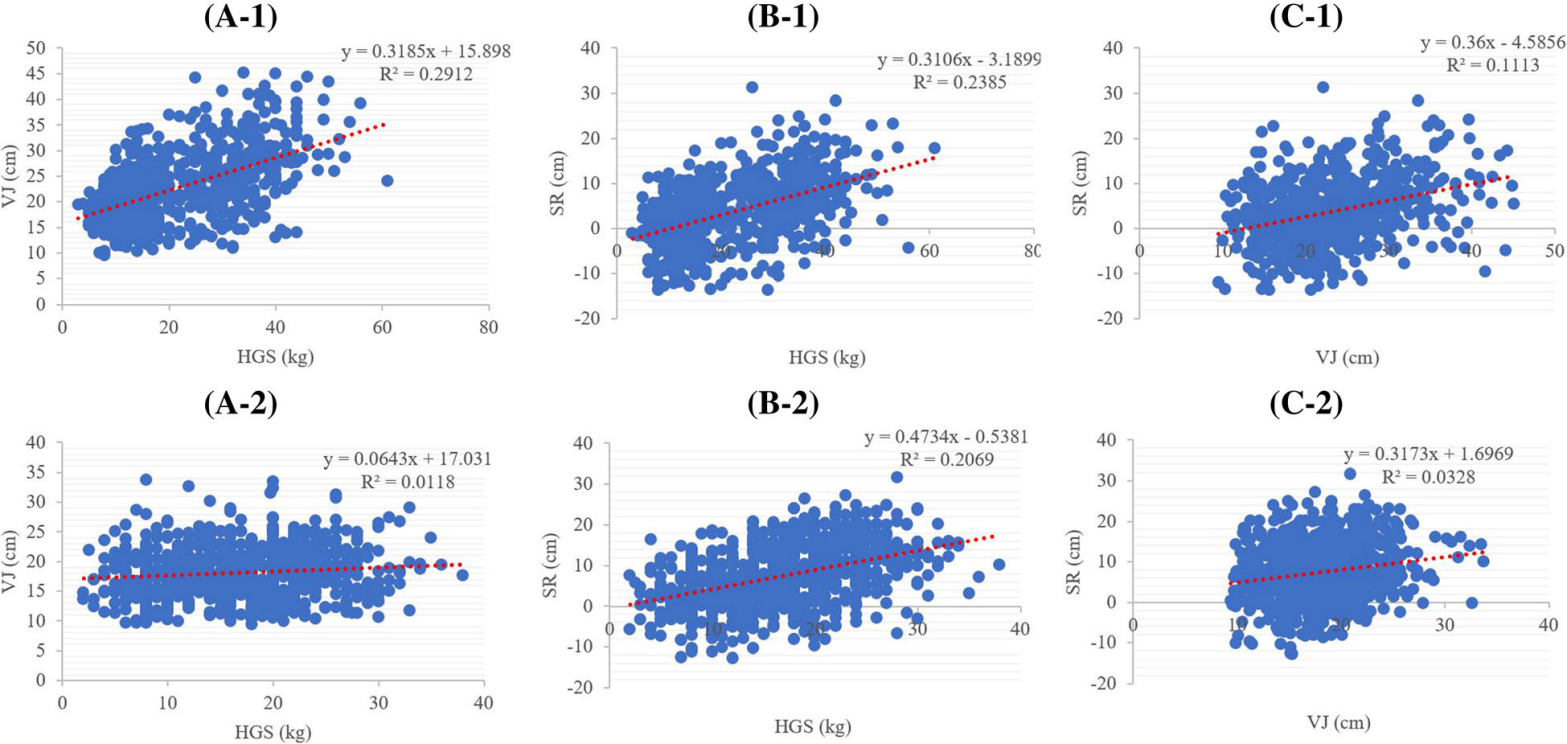
The relationship between muscle fitness indexes. **A-1**: relationship between GS and VJ in boys; **A-2**: relationship between GS and VJ in girls; **B-1**: relationship between GS and SR in boys; **B-2**: relationship between GS and SR in girls; **C-1**: relationship between VJ and SR in boys; **C-2**: relationship between VJ and SR in girls. GS: hand grip strength; VJ: vertical jump; SR: sit-and-reach

The correlation analyses suggested that all three MF indexes had significant mutual correlations (all *p* < 0.01) in both sexes. In boys, GS and VJ had the strongest correlation. By contrast, in girls, GS and VJ seemed to have a much weaker correlation, while still being statistically significant (*p* = 0.001). Details were presented in Fig. 3.

Furthermore, we compared the adjusted means of VJ in GS quartile groups. Similar comparisons of SR were also made to study the relationships among these three muscle fitness tests. Consistent with the correlation analyses, there were increasing trends for both VJ and SR with elevated GS quartiles in boys and girls (*p* < 0.001, Additional file 1: Figure S2).

## Discussion

To the best of our knowledge, this was the first study to investigate MF, measured using GS, VJ and SR, and its relationship with BMI in children and adolescents in China. As physical fitness and MF may play key roles in health in children and adolescents, it would be helpful to understand muscle function early in life and its relationship to BMI. This may be of value to understand the changes in the ability to have ideal function and health later in life.

By using a multi-stage stratified sampling method, we selected representative data reflecting BMI and MF among 7–18 years old children and adolescents. There were more participants recruited in Hainan than in Shaanxi Province because of longer recruitment time, better local government support and larger population size. Compared with Hainan, Shaanxi had more boys (51.87% vs. 42.60%) and more participants living in rural areas (63.08% vs. 40.27%) but their age distribution was similar (*p* = 0.588). Since we performed analyses by sex separately, and mostly adjusted by regions or urban/rural areas, the disproportion of socioeconomic characteristics in the two study sites would not cause severe bias in the study conclusions.

There were sex differences on BMI and MF in the study. Consistent with other relevant studies [20, 24], boys had higher stature in the same age group than girls, with the exception of the age groups below 10, and seemed to increase keep rising with similar rate through the 7–18 years stage. The stature of girls increased with age, but the rate fell after 11–12 years. Boys had higher prevalence of overweight/obesity than girls, especially in the ages 9–12.

China is a developing country with highly unbalanced regional development. In this study, regional and urban-rural disparities on BMI categories were identified. Compared with children and adolescents from Hainan Province, located in South China, subjects from Shaanxi Province had lower prevalence of thinness and higher prevalence of overweight/obesity. This sex and regional difference may be attributed to diversity of genomic backgrounds, physiology and environmental factors, such as different socioeconomic status (SES), nutrition status and physical exercises. Previous studies revealed that in developing countries, youths from a higher SES were more likely to be obese than youths from a lower SES [25, 26]. Studies have also shown that youths with higher SES and from urban areas were more likely to be obese than those from lower SES and from rural areas [27, 28]. Shaanxi province comprised more subjects from urban areas than that from Hainan, which may partially explain the higher prevalence of overweight/obesity and lower prevalence of thinness.

Based on our study, MF, except VJ, was found increased with age. In general, boys had higher GS and VJ until older age when GS and VJ values became similar to those of girls. Consistent with other studies, GS in boys accelerated specifically after the age of 12 [29, 30]. Compared with US children and adolescents (with an average performance value of 26.3 kg in the age 7 group and 79.7 kg in the age 15 group in boys) [18], Chinese youths had much lower GS value. This difference may be attribute to the vast disparity of genetic background and high prevalence of physical inactivity in Chinese children and adolescents [31].

There were limited data on the relationship between VJ and BMI, and thus its correlation with other MF indexes. Only a few studies described the VJ performance in certain populations, such as athletes or sports players [32, 33]. As a predictor of bone health, higher VJ may indicate a better status of bone mineral density [34]. The performance of SR, in which girls were of much greater performance than boys, indicated that girls may have better hamstring and hip flexibility [35]. Based on the result of logistic models, the regional difference disappeared after BMI was adjusted, implying that the original regional difference on MF was mainly caused by BMI disproportion between the two geographic areas.

The stratification analyses revealed that BMI may play key role in influencing MF. Increased BMI was positively associated with GS but normal weight group had the greatest performance of VJ and SR. Previous studies on US population and Taiwanese Chinese population had also observed the association between physical fitness and body weight. In Ervin’s study [18] on US population aged 6–15, results found that BMI was associated with strength. Studies on Taiwanese Chinese population showed that muscle strength and physical fitness were to be found associated with obesity [17, 36, 37]. However, our study was the first one in mainland China to explore the current situation of MF and its relationship with body weight.

In the sub-group analyses, BMI was associated with VJ and SR only in relatively younger age groups (Additional file 1: Table S6), which may imply that age could modify the association between BMI and muscle fitness. Further study with larger sample size needs to be done to clarify this possible modification.

The study had some limitations: Firstly, we did not assess maturation level of the participant, which may be a factor of great influence on fitness test and BMI. Secondly, the absence of detailed information on living environments, dietary patterns and physical activity limited our study on exploring other determinants of body weight and MF. Thirdly, we did not measure the test-retest reliability for SR, GS and VJ, and therefore we were not able to obtain the coefficient of variation for each. Fourthly, in Shaanxi Province, we only collected data on hand grip strength, which limited us to be able to study the regional difference on VJ and SR. Nonetheless, we were still able to use GS as a predictor for total muscle strength in children and adolescents [1]. The three indexes of MF were correlated with each other and the difference on GS among regions was representative to some extent to reveal geographic disparities on MF in the study population. Because dynamometer was more portable and the test of grip strength was much safer among elder population, hand grip strength test could be used as a feasible and important measurement for muscle strength in a much broader population. Lastly, we did not calculate the sampling weights because of the difficult to obtain the denominator, which was the total number of qualified participants in the survey areas. This may limit the generalizability to other studies.

## Conclusions

Our study was the first study to describe the current situation of MF status assessed by GS, VJ and SR in children and adolescents in China, with representative data for further exploration in other related study fields. It also explored the relationship between MF and BMI and found that increased body weight may have a positive association with isometric muscle strength measured by grip strength, but a negative one with strength of lifting the body. Sex difference was also found in the performance of flexibility. This study may provide evidence of the role of BMI on muscle fitness for clinicians and researchers based on the increasing prevalence of childhood obesity, as well as for policy makers to develop sex-specific strategies on body weight management and muscle performance promotion among children and adolescents in China.

## Additional file

Additional file 1: **Table S1–1.** The age and sex specific proportions of thinness, normal weight and overweight/obesity in Urban/Rural areas among participants aged 7–18, 2014. **Table S1–2.** The age and sex specific proportions of thinness, normal weight and overweight/obesity in different study sites among participants aged 7–18, 2014. **Table S2.** The age and sex specific means of hand grip strength stratified by BMI categories among children and adolescents aged 7–18, 2014. **Table S3.** The age and sex specific means of vertical jump stratified by BMI categories among children and adolescents aged 7–18, 2014. **Table S4.** The age and sex specific means of sit-and-reach stratified by BMI categories among children and adolescents aged 7–18, 2014. **Table S5.** The associated factors of BMI among participants aged 7–18 years in China, 2014. **Table S6.** The association between BMI and muscle fitness in children and adolescents aged 7–18 in China, stratified by age groups, 2014. **Figure S1.** Arithmetical means and deviations of hand grip strength, vertical jump and sit-and reach in sexes and age groups among children and adolescents aged 7–18, stratified by BMI categories. **Figure S2.** the adjusted means and standard errors of vertical jump and sit-and-reach, stratified by GS quartiles, in boys and girls aged 7–18 in mainland China. Covariates included residential areas and study sites, and BMI was adjusted using the LSMEANS statement in the GLM procedure in SAS. GS: hand grip strength; VJ: vertical jump; SR: sit-and-reach; Q: quartiles of grip strength. (DOCX 667 kb)

## Abbreviations

ANCOVA: Analysis of covariance
ANOVA: Analysis of variance
BMI: Body mass index
CI: Confidence interval
CVD: Cardiovascular disease
GLM: General linear regression model
GS: Hand grip strength
MF: Muscle fitness
OR: Odds ratio
SD: Standard deviation
SR: Sit-and-reach
VJ: Vertical jump
WHO: World health organization
