# Supplementary material for: Muscle fitness and its association with body mass index in children and adolescents aged 7–18 years in China: a cross-sectional study
Source: BMC Pediatr. 2019 Apr 10;19:101. doi: 10.1186/s12887-019-1477-8 (PMC6456985; doi:10.1186/s12887-019-1477-8)
Supplement: Supplementary file 1 — Table S1–1. The age and sex specific proportions of thinness, normal weight and overweight/obesity in Urban/Rural areas among participants aged 7–18, 2014. Table S1–2. The age and sex specific proportions of thinness, normal weight and overweight/obesity in different study sites among participants aged 7–18, 2014. Table S2. The age and sex specific means of hand grip strength stratified by BMI categories among children and adolescents aged 7–18, 2014. Table S3. The age and sex specific means of vertical jump stratified by BMI categories among children and adolescents aged 7–18, 2014. Table S4. The age and sex specific means of sit-and-reach stratified by BMI categories among children and adolescents aged 7–18, 2014. Table S5. The associated factors of BMI among participants aged 7–18 years in China, 2014. Table S6. The association between BMI and muscle fitness in children and adolescents aged 7–18 in China, stratified by age groups, 2014. Figure S1. Arithmetical means and deviations of hand grip strength, vertical jump and sit-and reach in sexes and age groups among children and adolescents aged 7–18, stratified by BMI categories. Figure S2. the adjusted means and standard errors of vertical jump and sit-and-reach, stratified by GS quartiles, in boys and girls aged 7–18 in mainland China. Covariates included residential areas and study sites, and BMI was adjusted using the LSMEANS statement in the GLM procedure in SAS. GS: hand grip strength; VJ: vertical jump; SR: sit-and-reach; Q: quartiles of grip strength. (DOCX 667 kb) [file 12887_2019_1477_MOESM1_ESM.docx]

**Additional file**

Table S1-1. The age and sex specific proportions of thinness, normal weight and overweight/obesity in Urban/Rural areas among participants aged 7-18, 2014.

|  | Urban | | | | | | | Rural | | | | | | |
| --- | --- | --- | --- | --- | --- | --- | --- | --- | --- | --- | --- | --- | --- | --- |
| Age | Thinness | | Normal weight | | Overweight/obesity | | Overall | Thinness | | Normal weight | | Overweight/obesity | | Overall |
| Boys | n | **%** | n | **%** | n | **%** | N | n | **%** | n | **%** | n | **%** | N |
| 7-8 | 0 | 0 | 10 | 83.33 | 2 | 16.67 | 12 | 8 | 13.11 | 50 | 81.97 | 3 | 4.92 | 61 |
| 9-10 | 2 | 2.33 | 56 | 65.12 | 28 | 32.56 | 86 | 19 | 15.20 | 97 | 77.60 | 9 | 7.20 | 125 |
| 11-12 | 11 | 9.82 | 76 | 67.86 | 25 | 22.32 | 112 | 25 | 21.37 | 85 | 72.65 | 7 | 5.98 | 117 |
| 13-14 | 10 | 9.71 | 83 | 80.58 | 10 | 9.71 | 103 | 15 | 19.23 | 59 | 75.64 | 4 | 5.13 | 78 |
| 15-16 | 9 | 10.98 | 71 | 86.59 | 2 | 2.44 | 82 | 17 | 22.97 | 51 | 68.92 | 6 | 8.11 | 74 |
| 17-18 | 18 | 15.13 | 97 | 81.51 | 4 | 3.36 | 119 | 12 | 19.67 | 46 | 75.41 | 3 | 4.92 | 61 |
| Girls |  |  |  |  |  |  |  |  |  |  |  |  |  |  |
| 7-8 | 3 | 15.79 | 13 | 68.42 | 3 | 15.79 | 19 | 9 | 12.00 | 61 | 81.33 | 5 | 6.67 | 75 |
| 9-10 | 7 | 7.45 | 74 | 78.72 | 13 | 13.83 | 94 | 33 | 20.63 | 118 | 73.75 | 9 | 5.63 | 160 |
| 11-12 | 14 | 13.08 | 84 | 78.50 | 9 | 8.41 | 107 | 19 | 16.81 | 92 | 81.42 | 2 | 1.77 | 113 |
| 13-14 | 6 | 7.41 | 71 | 87.65 | 4 | 4.94 | 81 | 7 | 11.48 | 52 | 85.25 | 2 | 3.28 | 61 |
| 15-16 | 15 | 8.52 | 156 | 88.64 | 5 | 2.84 | 176 | 9 | 14.52 | 52 | 83.87 | 1 | 1.61 | 62 |
| 17-18 | 13 | 5.78 | 208 | 92.44 | 4 | 1.78 | 225 | 7 | 8.97 | 70 | 89.74 | 1 | 1.28 | 78 |

Table S1-2. The age and sex specific proportions of thinness, normal weight and overweight/obesity in different study sites among participants aged 7-18, 2014.

|  | Shaanxi Province | | | | | | | Hainan Province | | | | | | |
| --- | --- | --- | --- | --- | --- | --- | --- | --- | --- | --- | --- | --- | --- | --- |
| Age group | Thinness | | Normal weight | | Overweight/obesity | | Overall | Thinness | | Normal weight | | Overweight/obesity | | Overall |
| Boys | n | **%** | n | **%** | n | **%** | N | n | **%** | n | **%** | n | **%** | N |
| 7-8 | 3 | 10.71 | 22 | 78.57 | 3 | 10.71 | 28 | 5 | 11.11 | 38 | 84.44 | 2 | 4.44 | 45 |
| 9-10 | 2 | 3.33 | 39 | 65.00 | 19 | 31.67 | 60 | 20 | 13.16 | 114 | 75.00 | 18 | 11.84 | 152 |
| 11-12 | 5 | 7.69 | 42 | 64.62 | 18 | 27.69 | 65 | 31 | 18.79 | 120 | 72.73 | 14 | 8.48 | 165 |
| 13-14 | 6 | 10.17 | 47 | 79.66 | 6 | 10.17 | 59 | 19 | 15.57 | 95 | 77.87 | 8 | 6.56 | 122 |
| 15-16 | 10 | 16.39 | 43 | 70.49 | 8 | 13.11 | 61 | 16 | 16.84 | 79 | 83.16 | 0 | 0 | 95 |
| 17-18 | 11 | 18.33 | 44 | 73.33 | 5 | 8.33 | 60 | 19 | 15.83 | 99 | 82.50 | 2 | 1.67 | 120 |
| Girls |  |  |  |  |  |  |  |  |  |  |  |  |  |  |
| 7-8 | 4 | 16.00 | 15 | 60.00 | 6 | 24.00 | 25 | 8 | 11.59 | 59 | 85.51 | 2 | 2.90 | 69 |
| 9-10 | 5 | 7.46 | 52 | 77.61 | 10 | 14.93 | 67 | 35 | 18.72 | 140 | 74.87 | 12 | 6.42 | 187 |
| 11-12 | 4 | 7.02 | 46 | 80.70 | 7 | 12.28 | 57 | 29 | 17.79 | 130 | 79.75 | 4 | 2.45 | 163 |
| 13-14 | 2 | 5.13 | 33 | 84.62 | 4 | 10.26 | 39 | 11 | 10.68 | 90 | 87.38 | 2 | 1.94 | 103 |
| 15-16 | 5 | 9.26 | 47 | 87.04 | 2 | 3.70 | 54 | 19 | 10.33 | 161 | 87.50 | 4 | 2.17 | 184 |
| 17-18 | 1 | 1.49 | 65 | 97.01 | 1 | 1.49 | 67 | 19 | 8.05 | 213 | 90.25 | 4 | 1.69 | 236 |

Table S2. The age and sex specific means of hand grip strength stratified by BMI categories among children and adolescents aged 7-18, 2014.

|  | Boys | | | | | | | | Girls | | | | | | | |
| --- | --- | --- | --- | --- | --- | --- | --- | --- | --- | --- | --- | --- | --- | --- | --- | --- |
| Age group | Thinness | | Normal weight | | Overweight/obesity | | Overall | | Thinness | | Normal weight | | Overweight/obesity | | Overall | |
|  | mean | SD | mean | SD | mean | SD | mean | SD | mean | SD | mean | SD | mean | SD | mean | SD |
| 7-8 | 6.63 | 2.45 | 9.72 | 2.37 | 10.00 | 1.22 | 9.40 | 2.49 | 6.96 | 2.67 | 7.26 | 1.99 | 8.88 | 2.36 | 7.36 | 2.14 |
| 9-10 | 10.45 | 2.42 | 11.71 | 3.19 | 13.35 | 3.60 | 11.88 | 3.29 | 8.03 | 2.39 | 9.86 | 3.10 | 11.36 | 2.75 | 9.70 | 3.08 |
| 11-12 | 12.46 | 2.88 | 16.01 | 5.26 | 17.35 | 5.58 | 15.62 | 5.19 | 10.70 | 2.59 | 14.91 | 4.52 | 18.82 | 7.33 | 14.47 | 4.80 |
| 13-14 | 18.32 | 5.28 | 24.84 | 6.57 | 30.17 | 9.99 | 24.29 | 7.19 | 15.85 | 2.85 | 18.76 | 4.59 | 19.83 | 2.93 | 18.54 | 4.47 |
| 15-16 | 26.15 | 7.00 | 34.19 | 6.99 | 33.13 | 8.01 | 32.79 | 7.61 | 18.63 | 4.30 | 20.89 | 4.39 | 26.00 | 5.93 | 20.79 | 4.53 |
| 17-18 | 32.40 | 6.21 | 37.75 | 6.59 | 39.14 | 4.63 | 36.91 | 6.75 | 21.10 | 5.89 | 22.66 | 4.64 | 23.80 | 6.61 | 22.58 | 4.76 |

Table S3. The age and sex specific means of vertical jump stratified by BMI categories among children and adolescents aged 7-18, 2014.

|  | Boys | | | | | | | | Girls | | | | | | | |
| --- | --- | --- | --- | --- | --- | --- | --- | --- | --- | --- | --- | --- | --- | --- | --- | --- |
| Age group | Thinness | | Normal weight | | Overweight/obesity | | Overall | | Thinness | | Normal weight | | Overweight/obesity | | Overall | |
|  | Mean | SD | mean | SD | mean | SD | mean | SD | mean | SD | mean | SD | mean | SD | mean | SD |
| 7-8 | 19.24 | 3.81 | 18.57 | 3.20 | 19.45 | 4.03 | 18.69 | 3.22 | 18.17 | 4.43 | 16.76 | 3.90 | 20.30 | 5.37 | 17.06 | 4.00 |
| 9-10 | 18.35 | 4.11 | 19.07 | 4.06 | 15.61 | 3.01 | 18.58 | 4.09 | 17.70 | 3.73 | 17.52 | 4.27 | 16.43 | 2.64 | 17.48 | 4.08 |
| 11-12 | 20.29 | 4.74 | 22.15 | 5.68 | 17.99 | 4.10 | 21.46 | 5.52 | 19.23 | 3.70 | 19.17 | 4.14 | 18.10 | 3.36 | 19.15 | 4.03 |
| 13-14 | 24.55 | 6.88 | 23.93 | 6.01 | 20.65 | 7.01 | 23.81 | 6.23 | 18.03 | 4.51 | 18.53 | 4.07 | 20.05 | 7.42 | 18.51 | 4.13 |
| 15-16 | 25.38 | 7.16 | 25.83 | 7.22 | - | - | 25.76 | 7.17 | 19.34 | 4.85 | 18.02 | 3.98 | 15.73 | 3.68 | 18.11 | 4.09 |
| 17-18 | 26.52 | 5.13 | 28.80 | 7.17 | 30.80 | 19.09 | 28.47 | 7.08 | 19.34 | 4.09 | 17.88 | 3.96 | 17.40 | 2.42 | 17.98 | 3.96 |

Table S4. The age and sex specific means of sit-and-reach stratified by BMI categories among children and adolescents aged 7-18, 2014.

|  | Boys | | | | | | | | Girls | | | | | | | |
| --- | --- | --- | --- | --- | --- | --- | --- | --- | --- | --- | --- | --- | --- | --- | --- | --- |
| Age group | Thinness | | Normal weight | | Overweight/obesity | | **Overall** | | Thinness | | Normal weight | | Overweight/obesity | | **Overall** | |
|  | mean | SD | mean | SD | mean | SD | mean | SD | mean | SD | mean | SD | mean | SD | mean | SD |
| 7-8 | -1.72 | 4.42 | 0.55 | 5.87 | -1.5 | 5.37 | 0.20 | 5.66 | 1.41 | 4.54 | 3.09 | 5.34 | 0.00 | 2.40 | 2.79 | 5.19 |
| 9-10 | 0.4 | 6.49 | 1.07 | 5.65 | -2.28 | 5.33 | 0.60 | 5.79 | 1.84 | 4.97 | 3.88 | 5.73 | 3.75 | 7.71 | 3.48 | 5.75 |
| 11-12 | -0.7 | 5.39 | 1.52 | 5.86 | 0.51 | 6.78 | 1.01 | 5.88 | 2.06 | 6.06 | 5.41 | 6.01 | 1.62 | 8.48 | 4.72 | 6.19 |
| 13-14 | 3.97 | 5.83 | 3.84 | 7.06 | 4.59 | 7.52 | 3.91 | 6.86 | 5.25 | 5.05 | 7.68 | 7.02 | 10.80 | 3.25 | 7.48 | 6.81 |
| 15-16 | 6.76 | 8.23 | 8.12 | 7.4 | - | - | 7.89 | 7.52 | 9.31 | 4.94 | 10.50 | 6.96 | 8.90 | 2.95 | 10.34 | 6.70 |
| 17-18 | 8.67 | 7.13 | 8.22 | 7.63 | 11.1 | 8.77 | 8.34 | 7.51 | 13.25 | 6.32 | 10.90 | 6.88 | 6.85 | 9.10 | 11.02 | 6.89 |

Table S5. The associated factors of BMI among participants aged 7-18 years in China, 2014.

|  | Thinness^*^ | | |  | Overweight/obesity^*^ | | |  |
| --- | --- | --- | --- | --- | --- | --- | --- | --- |
| Overall | *OR* | 95% *CI* | | *p* | *OR* | 95% *CI* | | *p* |
| Age | 1.006 | 0.964 | 1.049 | 0.790 | 0.816 | 0.769 | 0.865 | <0.001 |
| Sex (ref=girls) | 1.371 | 1.065 | 1.764 | 0.014 | 2.260 | 1.594 | 3.203 | <0.001 |
| Residential areas (ref=rural) | 0.447 | 0.336 | 0.594 | <0.001 | 3.118 | 2.164 | 4.492 | <0.001 |
| Shaanxi (ref=Hainan) | 0.534 | 0.387 | 0.737 | <0.001 | 3.688 | 2.615 | 5.202 | <0.001 |
| Boys |  |  |  |  |  |  |  |  |
| Age | 1.087 | 1.022 | 1.156 | 0.008 | 0.833 | 0.771 | 0.900 | <0.001 |
| Residential areas (ref=rural) | 0.381 | 0.251 | 0.576 | <0.001 | 3.550 | 2.223 | 5.670 | <0.001 |
| Shaanxi (ref=Hainan) | 0.541 | 0.348 | 0.841 | 0.006 | 3.935 | 2.526 | 6.132 | <0.001 |
| Girls |  |  |  |  |  |  |  |  |
| Age | 0.936 | 0.883 | 0.993 | 0.0272 | 0.800 | 0.729 | 0.877 | <0.001 |
| Residential areas (ref=rural) | 0.545 | 0.366 | 0.811 | 0.0028 | 2.557 | 1.424 | 4.591 | 0.0017 |
| Shaanxi (ref=Hainan) | 0.470 | 0.287 | 0.769 | 0.0027 | 3.394 | 1.960 | 5.877 | <0.001 |

^*^reference group=normal weight

Table S6. The association between BMI and muscle fitness in children and adolescents aged 7-18 in China, stratified by age groups, 2014.

| **Age group** | **BMI categories** | *B^a^* | *SE* | 95% *CI* | | *p* |
| --- | --- | --- | --- | --- | --- | --- |
| **Hand grip strength** | |  |  |  | |  |
| 7-10 | Thinness | 1.732 | 0.329 | -2.377 | -1.086 | <0.001 |
|  | Overweight/obesity | 1.623 | 0.355 | 0.927 | 2.320 | <0.001 |
| 11-14 | Thinness | -3.895 | 0.534 | -4.942 | -2.847 | <0.001 |
|  | Overweight/obesity | 2.025 | 0.707 | 0.640 | 3.410 | 0.004 |
| 15-18 | Thinness | -4.329 | 0.598 | -5.501 | -3.156 | <0.001 |
|  | Overweight/obesity | 1.901 | 1.116 | -0.286 | 4.089 | 0.089 |
| **Vertical jump** | |  |  |  |  |  |
| 7-10 | Thinness | -0.081 | 0.557 | -1.172 | 1.010 | 0.884 |
|  | Overweight/obesity | -1.399 | 0.763 | -2.895 | 0.096 | 0.067 |
| 11-14 | Thinness | -0.318 | 0.612 | -1.518 | 0.882 | 0.604 |
|  | Overweight/obesity | -3.281 | 1.024 | -5.287 | -1.275 | 0.001 |
| 15-18 | Thinness | 0.083 | 0.678 | -1.246 | 1.412 | 0.903 |
|  | Overweight/obesity | 0.509 | 1.693 | -3.827 | 2.810 | 0.764 |
| **Sit-and-reach** | |  |  |  |  |  |
| 7-10 | Thinness | -1.826 | 0.753 | -3.302 | -0.350 | 0.015 |
|  | Overweight/obesity | -1.422 | 1.086 | -3.551 | 0.706 | 0.190 |
| 11-14 | Thinness | 1.864 | 0.732 | -3.299 | -0.428 | 0.011 |
|  | Overweight/obesity | -0.800 | 1.245 | -3.241 | 1.641 | 0.521 |
| 15-18 | Thinness | 0.157 | 0.889 | -1.585 | 1.898 | 0.860 |
|  | Overweight/obesity | -1.738 | 2.246 | -6.139 | 2.663 | 0.439 |

^a^The results were adjusted for age, sex, residential areas and study sites (only for hand grip strength), and the normal weight group was set as reference. Age was analyzed as continuous data; sex, BMI, residential areas and study sites were set as dummy variables. BMI: body mass index (kg/m^2^). B: regression coefficient; SE: standard error of regression coefficient; CI: confidence interval.

Figure S1


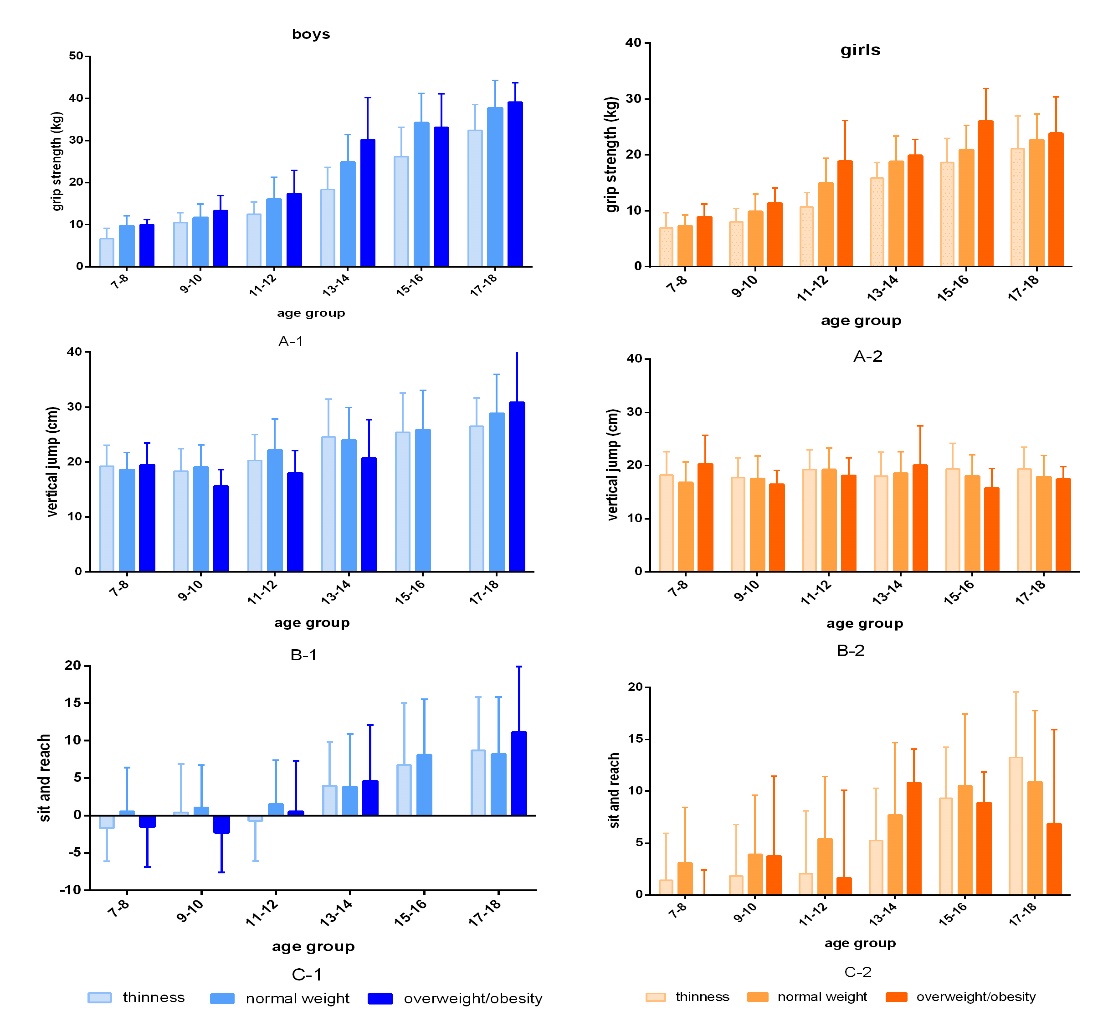


Figure S1. [Arithmetical](https://cn.bing.com/dict/clientsearch?mkt=zh-CN&setLang=zh&form=BDVEHC&ClientVer=BDDTV3.5.1.4320&q=%E7%AE%97%E6%9C%AF%E5%9D%87%E6%95%B0) [mean](https://cn.bing.com/dict/clientsearch?mkt=zh-CN&setLang=zh&form=BDVEHC&ClientVer=BDDTV3.5.1.4320&q=%E7%AE%97%E6%9C%AF%E5%9D%87%E6%95%B0)s and deviations of hand grip strength, vertical jump and sit-and reach in sexes and age groups among children and adolescents aged 7-18, stratified by BMI categories.

Figure S2.

Figure S2. the adjusted means and standard errors of vertical jump and sit-and-reach, stratified by GS quartiles, in boys and girls aged 7-18 in mainland China. Covariates included residential areas and study sites, and BMI was adjusted using the LSMEANS statement in the GLM procedure in SAS. GS: hand grip strength; VJ: vertical jump; SR: sit-and-reach; Q: quartiles of grip strength.
